# Surgical resection due to poor outcome of the immunotherapy of a relapsed mediastinal liposarcoma: a case report

**DOI:** 10.2144/fsoa-2023-0099

**Published:** 2024-05-15

**Authors:** Ming-Ji Wang, Shu-Quan Xu, Lei-Lei Wu, Zhi-Xin Li, Dong Xie

**Affiliations:** 1 Department of Thoracic Surgery, Fuqing City Hospital Affiliated to Fujian Medical University, Fuqing, PR China; 2 School of Medicine, Tongji University, Shanghai, PR China; 3 Department of Thoracic Surgery, Shanghai Pulmonary Hospital, Tongji University School of Medicine, Shanghai, PR China

**Keywords:** liposarcoma, palbociclib, pembrolizumab, second surgery

## Abstract

The feasibility of surgery after immunotherapy for mediastinal liposarcoma remains uncertain. Besides, the case of immunotherapy for liposarcoma is still lacking. We report a case of recurrence after resection of a left mediastinal liposarcoma. After recurrence, one course of pembrolizumab plus anlotinib hydrochloride showed no tumor shrinkage, and genetic testing showed CDK4 amplification and PD-L1 TPS <1%; therefore, the plan was changed to one course of pembrolizumab plus palbociclib, but the tumor still did not shrink. Thus, second tumor resection was performed. In addition, the postoperative pathology was still well-differentiated liposarcoma. The significance of immunotherapy in liposarcoma still needs to be further explored. In the absence of surgical contraindications, secondary surgery might be feasible.

## Abstract (plain language summary)

This case with CDK4 amplification and PD-L1 TPS <1% responds weakly to the combined immunotherapy after the surgery.

The significance of immunotherapy in liposarcoma still needs to be further explored.

In the absence of surgical contraindications, secondary surgery is feasible.

Indicators to predict the effectiveness of immunotherapy still need to be explored and confirmed in the liposarcoma.

Liposarcoma is one of the most common malignancies of soft tissue, including four basic types: well-differentiated/atypical lipomatous tumor (WDL/ALT), dedifferentiated, myxoid and pleomorphic liposarcoma, recognized by the World Health Organization (WHO). WDL/ALT is the most common type of liposarcoma in the mediastinum, especially for middle-aged to elderly adults [1]. The surgery as the first-line treatment of liposarcoma is accompanied with the problem that patients with WDL have a high tendency to develop a local postoperative recurrence [2]. Especially for infrequent retroperitoneal liposarcoma, the method to prevent recurrence is important. The diagnosis of well-differentiated retroperitoneal liposarcoma and postoperative follow-up of patients is very difficult. Shorter follow-up interval with the computer tomography (CT) or the magnetic resonance imaging (MRI) would be helpful for early detection [3]. The anthracycline-based chemotherapy has been widely recommended to be added in first-line therapy for liposarcoma, which achieve a response at the cost of higher toxicity and no statistically significant benefit for overall survival [4]. As clinical medicine enters the era of individualized treatment and precision therapy, the role of immunotherapy and targeted therapy is becoming more and more important. For example, immunotherapy in non-small cell lung cancer and esophageal squamous cell carcinoma has been proved to prolong the progression-free survival and overall survival [5]. For infrequent soft tissue sarcoma, the systemic management performed by experienced doctors has benefited the patients much [6]. Immunotherapy, an emergent and potentially promising therapy for tumors, has rarely been reported for liposarcoma. According to a phase II trial of pembrolizumab in advanced soft-tissue sarcoma, the response of liposarcoma patients is only 20% [7]. We now report a case of liposarcoma in mediastinum with combination immunotherapy, hoping it can help the clinical practice.

## Results

**Figure 1.**
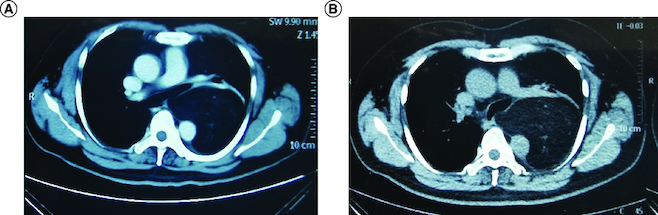
CT images of the tumor. CT picture before first surgery **(A)** and recurrence CT picture after first surgery **(B).**

A male patient was diagnosed with liposarcoma of the left mediastinum when he was 49 years old. The CT showed lamellar density shadow of middle and posterior mediastinum at the size of 10.6*8.9 cm, and no obvious enhancement was found in enhanced CT (Figure 1A). The left main bronchus and descending aorta were oppressed and displaced, accompanied by the compression of the left lower lobar bronchus (Figure 1A). Then, the patient received surgical treatment. The postoperative pathology indicated WDL and negative surgical margins. No adjuvant therapy was administered for the patients after resection. The patient received regular CT scans at 6-month intervals in the outpatient clinic.

**Figure 2.**
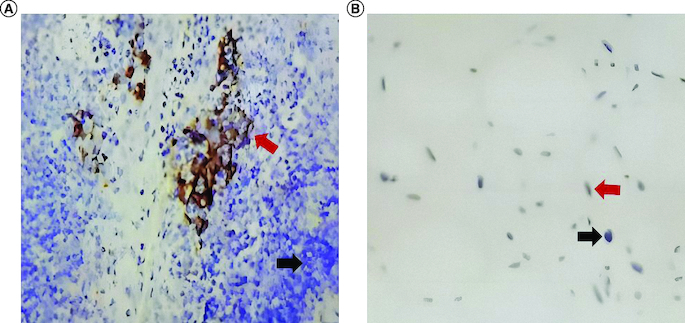
Immunohistochemistry images of the tumor. Immunohistochemistry for control tissue **(A)** and liposarcoma of this patient **(B).** Black arrows indicate negative staining, and red arrows indicate positive staining.

Unfortunately, a left mediastinal mass was observed in the chest CT 12 months after surgery, which showed a little fat density shadow in the middle mediastinum at the size of 9.7*6.6*11.9 cm (Figure 1B). The molecular test of the postoperative paraffin tissue was performed, and the results showed that the tumor cell proportion score (TPS) of the PD-L1 was smaller than 1% (Figure 2). Then, the patient received a course of pembrolizumab plus anlotinib hydrochloride combination therapy. After 21 days, he received a chest CT, which showed the size of the mass was 10.5*7.7*12.7 cm, and the tumor did not shrink. At the same time, the next-generation sequencing was conducted in the paraffin tissue of this patient and showed that the gene of CDK4 was amplification. Therefore, the treatment changed to the pembrolizumab (200 mg) intravenous drip plus palbociclib (100 mg) taken orally one tablet per day, day 1–21. No obvious shrinkage of the tumor was observed in the CT scan after 1 month.

Thus, we performed the second operation for this patient. Before the operation, we conducted a complete preoperative examination. The summary results were as follows: red blood cell 4.51 × 10^12^/l; white blood cell 6.86 × 10^9^/l; neutrophil 4.60 × 10^9^/l; platelets 301 × 10^9^/l; aspartate transaminase 13 IU/l; alanine aminotransferase 24 IU/l; albumin 42 g/l; globulin 25 g/l; blood urea nitrogen 3.8 mmol/l; creatinine 65 umol/l; uric acid 384 umol/l, all of which showed no problem. Besides, considering the size of the tumor, cardiac ultrasound and bronchoscopy were also necessary. The results of the echocardiogram indicated the normal size of the atrium and ventricle, and the left ventricular ejection fraction was 61%. Because of the large size of the mediastinal liposarcoma, we observed it pressed the left lower lobe in the CT scan, and bronchoscopy also showed corresponding results. We eliminated contraindications of operation through preoperative examination. After a multidisciplinary discussion, we decided to perform a second operation. The tumor was located in the middle and posterior mediastinum, with the longest diameter of about 15 cm, compressing the left lower lung, esophagus and thoracic aorta. Adhesions surrounded the tumor, but the boundary between the tumor and the surrounding tissue was still clear. The whole operation took 275 min, and the intraoperative bleeding was about 400 ml. Fortunately, intraoperative pathology showed negative surgical margins. The results of the chest x-ray were reviewed one month after surgery and suggested that the mediastinum presented postoperative changes; besides, the thorax was symmetrical; the texture of both lungs was increased, with no obvious abnormal density shadow was seen; the morphology and size position of the pulmonary hilum was not abnormal; the mediastinum was centered; both diaphragms were bright, and the diaphragmatic angles of both ribs were sharp; no significant abnormalities were seen in the heart shadow.

The results of the postoperative pathological diagnosis showed that the tumor was still well-differentiated liposarcoma. In addition, the immunohistochemistry showed that CDK4(+), KI67(1%+), MDM2(-), P16(+), S100(+), P53(-), CD34(+). This patient did not receive any adjuvant therapy after second surgery. However, the tumor was recurrence 12 months after second surgery. The follow-up time was updated to 20 September 2022. We connected the patient through a social networking site (WeChat). The patient still had CT scans every 3 months to observe the changes of the tumor size and waited for the next treatment.

## Discussion

In the present study, the patient received two operations and immune therapy before the second surgery. Regrettably, the immune therapy did not provide the patient with the benefit of tumor regression. In addition, liposarcoma is a malignancy that is easy to relapse locally. Therefore, the patient had to receive a second surgery. Unfortunately, 12 months after the surgery, the tumor recurred. Now, the patient still had CT scans every 3 months to observe the changes in the tumor size and waited for the next treatment. The chemotherapy regimens and radiation treatment remain viable adjuncts; surgical treatment with complete tumor excision (as stated by negative surgical margins) remains the gold standard. According to the literature, there is a significant difference in mid-term survival rates for patients with adequate tumor excision compared with excision with positive surgical margins, which further supports surgical treatment as the most effective treatment option [8]. However, performing a third surgery, or opting for pharmacological treatment can be a challenge for clinical practice.

The failure of anti-PD-1 immunotherapy, as we reported, is not an unpredictable event in liposarcoma with pembrolizumab. The existing results of pembrolizumab's response to liposarcoma have a significant feature – PD-L1 TPS >1% [7]. Moreover, the higher PD-L1 expression has a more improved clinical outcome [9]. By contrast, in our case, the PD-L1 TPS is lower than 1%, which may cause the inefficiency of the pembrolizumab.

Meanwhile, we should figure out that the results of pembrolizumab monotherapy are not reliable enough to be used in the clinic [7]. However, the incidence of liposarcoma is rare; therefore, the number of studies on liposarcoma is relatively small [10]. For this reason, although immunotherapy has proven its effectiveness in other solid tumors (such as non-small cell lung cancer), its significance in liposarcoma still needs to be further explored. For now, a phase II Study of Eribulin and Pembrolizumab in soft tissue sarcomas (NCT03899805) has been in progress. Of note, the criteria of this clinical trial do not include the molecular test for immune therapy-related biomarker, such as the expression of PD-L1, TPS and tumor mutation burden. According to the outcomes of other clinical trials, the combination of chemotherapy and immunotherapy is better than immunotherapy alone [11]. Therefore, the result of a combination of chemotherapy and immunotherapy in liposarcoma is worth to wait.

In this case, we used not only an immunotherapy drug but also a targeted drug, the CDK4 inhibitor, palbocilib. It has been shown that CDK4 is amplified in over 90% of retroperitoneal WDL/DDL and is a potential therapeutic target for liposarcoma, however the CDK4 inhibitor clinical efficacy has not been demonstrated [12]. Progression-free survival in patients treated with the CDK4 inhibitor palbocilib (PD0332991) has been shown to be superior to other second-line agents in existing phase II clinical trials [13]. However, in our case, palbocilib did not achieve the expected efficacy. In our analysis, CDK4 inhibitor resistance may have occurred. Mechanisms such as RB gene deletion play an important role in the mechanism of CDK4 inhibitor resistance [14]. However, we did not detect CDK4 resistance-related genes. Therefore, the clinical use and resistance of palbocilib in this case warrants further investigation.

In the field of efficiency evaluation of immunotherapy, we are short of a gold standard to evaluate the prognosis. The Food and Drug Administration (FDA) approved pembrolizumab for the first-line treatment of patients with a PD-L1 TPS of 50% or greater and pembrolizumab plus platinum combination therapy in patients regardless of PD-L1 expression. We still require a more efficient standard, considering the high expense of immunotherapy for the patient. Pretreatment PD-L1 expression and tumor mutation burden (TMB) in resectable non-small cell lung cancer has been proven significant benefits for patients [15]. For now, 18F-FDG PET/CT has been widely used in clinical practice for the evaluation of response to therapies in patients with lung cancer [16]. The immune-related gene prognostic index based on GEO database and real-time PCR technology may be used as a potential biomarker to evaluate the response and efficacy of immunotherapy in papillary renal cell carcinoma [17]. With the progress of cell and molecular biology, more detection indicators deserve attention.

There are still some limitations in this study. First, this is a case report, which can only provide some informative information to clinicians and cannot represent the outcome that immunotherapy is doomed to fail in treating all patients. Thus, we need more cases to perform a retrospective or prospective study to confirm our findings. Second, secondary surgery depends on the patient's physical condition, the adhesions around the tumor, and the closeness of the tumor to the surrounding tissues and organs. Therefore, the second operation is not universal but gives clinicians and patients one more treatment option to consider. Third, we cannot provide the overall survival time, as the observational end point is not reached.

## Conclusion

The significance of immunotherapy in liposarcoma still needs to be further explored. In the absence of surgical contraindications, secondary surgery might be feasible.
